# Decreased Reward Sensitivity in Rats from the Fischer344 Strain Compared to Wistar Rats Is Paralleled by Differences in Endocannabinoid Signaling

**DOI:** 10.1371/journal.pone.0031169

**Published:** 2012-02-08

**Authors:** Theresa Brand, Rainer Spanagel, Miriam Schneider

**Affiliations:** 1 Research Group Developmental Neuropsychopharmacology, Institute of Psychopharmacology, Central Institute of Mental Health, Medical Faculty Mannheim, University of Heidelberg, Mannheim, Germany; 2 Institute of Psychopharmacology, Central Institute of Mental Health, Medical Faculty Mannheim, University of Heidelberg, Mannheim, Germany; Duke University, United States of America

## Abstract

**Background:**

The aim of the present study was to examine if differences in the endocannabinoid (ECB) system might be linked to strain specific variations in reward-related behavior in Fischer344 (Fischer) and Wistar rats.

**Methodology/Principal Findings:**

Two rat strains, the Fischer and the Wistar strain, were tested for different aspects of reward sensitivity for a palatable food reward (sweetened condensed milk, SCM) in a limited-access intake test, a progressive ratio (PR) schedule and the pleasure-attenuated startle (PAS) paradigm. Additionally, basic differences in the ECB system and cannabinoid pharmacology were examined in both rat strains. Fischer rats were found to express lower reward sensitivity towards SCM compared to Wistar rats. These differences were observed for consummatory, motivational and hedonic aspects of the palatable food reward. Western blot analysis for the CB1 receptor and the ECB degrading enzyme fatty acid amide hydrolase (FAAH) revealed a lower expression of both proteins in the hippocampus (HPC) of Fischer rats compared to the Wistar strain. Furthermore, increased cannabinoid-stimulated extracellular-regulated kinase (ERK) phosphorylation was detected in Wistar rats compared to the Fischer strain, indicating alterations in ECB signaling. These findings were further supported by the pharmacological results, where Fischer rats were found to be less sensitive towards the effects of the CB1 receptor antagonist/inverse agonist SR141716 and the cannabinoid agonist WIN 55,212-2.

**Conclusions/Significance:**

Our present findings indicate differences in the expression of the CB1 receptor and FAAH, as well as the activation of ECB signaling pathways between Fischer and Wistar rats. These basic differences in the ECB system might contribute to the pronounced differences observed in reward sensitivity between both rat strains.

## Introduction

Cross-strain comparisons have provided a productive approach to understanding behavioral traits and their underlying neurobiological substrates in rodents. Already in the 1920s it had been discovered that rodent strains differ in their behavior [1] and since then many studies have attempted to correlate behavioral differences with genetic and neurobiological variables [2]. In particular for emotional behavior it is already well established that anxiety- and stress-responsiveness in rodents are highly influenced by the background strain which has been shown in a variety of different studies [3]–[7]. Notably, the role of strain differences in the context of reward-related behaviors is less well studied, especially for natural rewards. Although various studies indicate pronounced rat strain differences in the behavioral and molecular response to drugs of abuse, such as opioids, psychostimulants or ethanol [8]–[13], only little is known about similar strain differences on natural rewards, such as palatable food. Rat strain differences have been reported for opioid-induced feeding behavior, with Lewis rats showing a higher feeding responses to morphine than Fischer344 (Fischer) rats [14]. Kearns et al. [15] found that Lewis rats acquire autoshaping procedures in an operant task for food more rapidly and also perform at a higher response rate than Fischer rats.

One important neurotransmitter system that is strongly implicated in the modulation of reward processing for drug and non-drug rewards is the endocannabinoid (ECB) system [16]–[19]. However, few studies have investigated if alterations in this important modulatory system might be involved in strain specific behavioral variances. One interesting study demonstrated that Lewis rats are less sensitive to the behavioral, physiological and neural effects of cannabinoids than Wistar rats, showing mainly differences in c-fos expression, which were paralleled by behavioral findings [20]. Additionally, a cannabinoid receptor agonist was found to induce a stronger effect on anxiety-related behaviors in the Wistar strain compared to Lewis rats [21]. Further studies were mostly restricted to the rewarding or euphoric effects of cannabinoids in different rat strains. It has been demonstrated that Fischer rats show a lower responsiveness towards the effects of Δ^9^-tetrahydrocannabinol (THC) on intracranial self stimulation [22], [23]. Furthermore, strain differences have also been reported on the acquisition and stable performance of cannabinoid self-administration behavior [24]. These findings on strain differences in reward-related behaviors upon cannabinoid treatment are consistent with other studies demonstrating a high variety of general sensitivity towards drugs of abuse among different rat strains [8], [10], [13], [25]. Therefore, it is not unlikely that basic genetic differences in the ECB system might be involved in strain specific differences in reward perception and sensitivity.

The aim of the present study was to examine basic differences in reward-related behavior towards a palatable food reward in two different rat strains. Additionally, differences in ECB signaling and cannabinoid pharmacology were also investigated. For this study we chose the outbred Wistar-Han (Wistar) strain and the inbred Fischer strain, since both strains are well known to differ highly in their emotional responsiveness [26], [27], but have not been compared so far in the context of reward-related behavior. All animals were tested for reward sensitivity for a palatable food reward (limited free sweetened condensed milk (SCM) consumption, progressive ratio (PR) performance and in the pleasure attenuated startle (PAS) paradigm) and for their behavioral response towards the CB1 receptor antagonist/inverse agonist SR141716 (SR) and the cannabinoid agonist WIN 55,212-2 (WIN). Additionally, protein levels of the CB1 receptor and the principal anandamide (AEA) degrading enzyme fatty acide amide hydrolase (FAAH) as well as cannabinoid-induced stimulation of extracellular signal-regulated kinase-1 and -2 (ERK1/2), a mitogen-activated protein kinase, were examined by Western Blot analysis in different brain regions involved in reward processing.

## Materials and Methods

### Ethics Statement

All experiments were done in accordance with the NIH ethical guidelines for the care and use of laboratory animals for experiments, and were approved by the Regierungspräsidium Karlsruhe (Referat 35, Karlsruhe, Germany, 35-9185.81/G-56/07).

### Subjects

A total of 93 animals were used for the present study. Male adult Wistar Han™ (Wistar) and Fischer344/NHsd (Fischer) rats were purchased from Harlan Winkelmann GmbH (Borchen, Germany). Animals were housed in the same room in Makrolon™ cages (Eurostandard type IV) in groups of 6 on a 12 h light-dark schedule (lights on 8:00–20:00). During all experiments animals had free access to tap water and standard lab food, except for the PAS experiment, where animals were maintained on approximately 95% of their free-feeding bodyweight during testing and training. Before testing started, animals were allowed to recover from transportation and were habituated to the new environment and the experimenter for at least 7 days after arrival.

### Drugs

The CB1 receptor agonist WIN 55,212-2 (WIN) (Sigma-Aldrich, Munich, Germany) was freshly dissolved in Tween80 (Sigma-Aldrich, Munich, Germany) and diluted in Saline (0.9%) (Fresenius Kabi, Bad Homburg, Germany). For stimulation of phospho-ERK (pERK), vehicle (Tween80 and Saline) or WIN (2 mg/kg), were administered intraperitoneally 1 hour prior to decapitation with an injection volume of 1 ml/kg. For open field testing, vehicle (Tween80 and Saline) or WIN (2 mg/kg) were injected 10 min prior to behavioral testing. The CB1 receptor antagonist/inverse agonist SR141716 (SR; Rimonabant) (generously provided by NIMH) was dissolved in ethanol and Tween80 and diluted with saline. Three different concentrations were used for pharmacological experiments: 0.3, 0.6 and 1.2 mg/kg. SR was injected 30 min prior to testing with an injection volume of 1 ml/kg.

### Experimental design

For the present study three different cohorts of Fischer and Wistar rats were used. The first group (Fischer and Wistar: n = 12) was used for testing of reward-related behaviors. All animals were tested for SCM intake, followed by performance in a PR task and the PAS paradigm. Animals were left undisturbed for 1 week between the different behavioral tasks. After cessation of behavioral testing some of these animals (Fischer and Wistar: n = 8) were further used to examine the effects of WIN in an open field. The second cohort of animals (Fischer: n = 11, Wistar: n = 10) was used for pharmacological testing where the effects of the CB1 receptor antagonist/inverse agonist SR on SCM intake were investigated. Finally, a third group of naive rats was used for molecular analysis (CB1: Fischer and Wistar: n = 6, FAAH: Fischer and Wistar: n = 6, pERK: Fischer and Wistar: n = 12). For pERK stimulation, 6 rats of each strain received the vehicle injection and the rest were treated with 2 mg/kg WIN.

Since it is well known that Fischer rats are more anxious than Wistar rats [4], [26], [27], all animals were habituated extensively to experimental conditions and procedures in order to exclude possible novelty or stress effects.

### Behavioral Testing

#### Sweetened Condensed Milk (SCM) Intake

SCM (Leche Condensada, La Lechera from Nestle Barcelona, Spain) was freshly mixed 1∶3 with water on the day of use. All rats were habituated once in their homecage for 24 h to the SCM, 72 h prior to testing as previously described [28]. The test was conducted in single cages (Makrolon™, Eurostandard type III) and in order to avoid novelty-induced hypophagia animals were additionally habituated for two consecutive days to the single cages and SCM presentation in these single cages. On the test day, the body weight was measured and animals were placed in the single test cages. After an inital cage habituation time of 5 minutes, they received free access to a bottle of SCM for 15 minutes. SCM intake was then calculated as g intake per kg body weight (BW).

#### Progressive Ratio (PR) testing for SCM

PR test and training were conducted in an operant chamber (30.5 cm×24.1 cm×21.0 cm from Med Associates Inc., St. Albans, USA), which was controlled by the computer program MED-PCIV (Med Associates Inc.) as previously described [29]. During the first three days rats were habituated for 20 min daily to the operant chamber with free access to SCM (shaping). After shaping, rats were trained for lever-pressing in sessions of 30 minutes. Pressing the lever was paired with access to 90 µl SCM (reward) and a light signal. Training was performed under continuous reinforcement (CRF) until a stable baseline was reached (at least 60 lever presses per session). Rats not accomplishing this criterion at least 2 days prior to the PR test were excluded. After lever-response training was completed, one PR session (for 30 minutes) was conducted on the subsequent day. In that test the number of lever presses required to obtain a reward increased sequentially according to a PR2 progression: 1, 2, 4, 6, 8, 10, ‥, . During PR testing three behavioral measures were recorded. First we recorded the total number of lever presses and also the highest completed PR sequence during the 30 minute test session. Finally, the inactivity ratio, which is defined as the final completed sequence before the animals ceased responding for more than 2 minutes, was used to determine the occurrence of the ‘break point’ in PR performance. This break point is considered an operational measure for a shift in motivation, where the rewarding value is lower than the effort the animal is willing to invest to obtain this reward (Schneider et al. 2010).

#### Pleasure-attenuated startle (PAS)

Startle testing occurred in a startle chamber (SR-LAB; San Diego Instruments, San Diego, USA) that has been previously described in detail [28], [29]. A white noise pulse was used as the startle stimulus, with duration of 40 ms and an intensity of 100 dB sound pressure level (SPL) in Wistar rats and 115 dB SPL in Fischer rats. Preliminary experiments indicated Fischer rats showing a much lower startle reaction than Wistar rats, or even no response at all, when tested with the same startle intensity. We therefore performed a series of initial experiments in order to detect the startle stimulus intensity at which Fischer rats show a robust acoustic startle response (ASR) amplitude (data not shown), which was found to be 115 dB. An acclimatization time of 5 min, during which the rats received no stimulus except for the background noise (60 dB), was followed by the presentation of 5 initial startle stimuli. The test protocol consisted of 30 startle pulses with an inter-trial interval randomized between 10 and 20 s. Animals were tested twice for their ASR in the presence of an odor cue (orange, essential oils, Primavera Life, Sulzberg, Germany), once prior ( = ASR baseline; ASR1) and again 24 hours after 5 days of odor-reward association training (ASR2). The odor (30 µl) was provided in a petri dish that was placed in the box during habituation. PAS was calculated as mean percent decrease over baseline ASR amplitude [100−(100×mean ASR2 amplitude/mean ASR1 (baseline) amplitude)], as previously described. Reward association training lasted 5 days. During training, lasting 90 min in total, rats were placed in single cages (Eurostandard type III) and experienced 3 odor-reward presentations at random time points. The odor (orange, 15 µl) was supplied in a small Petri dish containing a piece of filter paper that was placed in the middle of the wire lid, 2 cm beneath the aperture of the SCM drinking bottle.

### Pharmacology

#### Effects of WIN on locomotor activity

In order to assess basic differences in cannabinoid pharmacology between the two rat strains, we tested the effects of a high dose of WIN (2 mg/kg) on locomotor activity in an open field. The open field consisted of four equal areas (51 cm×51 cm×50 cm) made of dark PVC. Distance traveled [cm] was digitally recorded in the open field apparatus for 30 minutes. The test was started by placing the rats in the center of the box. For the analysis of locomotor activity the observation program Viewer^2^ (Biobserve GmbH, Bonn, Germany) was used.

#### Effects of SR on SCM intake

The effects of the CB1 receptor antagonist/inverse agonist SR were tested with a within-subject design. We have shown before that SCM intake in adult rats can be measured repeatedly without any habituation effect [30]. Therefore, animals were tested four times in the limited access SCM intake paradigm (every 10^th^ day) and received on each day of testing an injection of either vehicle or SR (0.3, 0.6 or 1.2 mg/kg) which was randomized according to a latin square. SCM intake was then calculated as g intake per kg BW and additionally the percentage reduction of SCM intake after SR injections was calculated for each animal.

### Molecular Analysis

#### Rat Brain Preparation and Dissection

Rats were deeply anesthetized with a mixture of air and carbon dioxide (CO_2_) and sacrificed by decapitation. Brains were removed quickly and the following brain regions were dissected: medial prefrontal cortex (mPFC), striatum, nucleus accumbens (NAC) and hippocampus (HPC). Dissected brain tissues were homogenized in 1 ml lysis buffer (10 mM TrisHCl, 2 mM EDTA, pH 8.0) containing protease inhibitors (Roche Diagnostics, Penzberg, Germany) on crushed ice with a glass homogenizer.

#### Western Blot Analysis of FAAH and CB1 Receptor

The protein content was measured by the Bradford Protein Assay (BioRad Laboratories GmbH, Munich, Germany) using bovine serum albumin (Sigma-Aldrich, Munich, Germany) as standard, and amounts equivalent to 25 µg of protein of the brain region were electrophoresed in NuPAGE® Novex Bis-Tris Mini Gel 4–12% gel (Invitrogen, Darmstadt, Germany) after mixing with 4X Laemmli's Sample Buffer and heating up to 95°C for 5 minutes. Rainbow colored molecular weight marker (GE Healthcare, Munich, Germany) was included on each gel.

The electrophoresis was carried out at 200 V at room temperature and proteins were then blotted onto PVDF membranes (BioRad Laboratories GmbH, Munich, Germany) in tris/glycine/ethanol transfer buffer at 400 mA for 90 minutes. The membranes were then incubated with a rabbit polyclonal antibody against FAAH (amino acids 561–579, C-terminal)(1∶1000, Enzo Life Sciences AG, formerly ALEXIS corporation, Lausen, Switzerland; Cat. No. ALX-210-418) at 4°C over night in 5% non-fat dry milk solution, followed by HRP (horseradish-peroxidase)-linked anti-rabbit (1∶2000 Cayman Chemical, Ann Arbor, USA) for 1 hour at room temperature in 2.5% nonfat dry milk solution and developed by chemiluminescence (ECL, GE Healthcare, Munich, Germany). Same blots were washed in water and tris-buffered saline and incubated with monocolonal anti-ß-Actin (1∶2000 New England Biolabs GmbH, Frankfurt, Germany), followed by HRP-linked anti-rabbit and visualization by ECL. A similar procedure was used for the analysis of CB1 receptor levels in brain samples, using the polyclonal anti-CB1 receptor antibody (against amino acids 1–14 of the CB1 Receptor; N-terminal)(1∶1000, Cayman Chemical, Ann Arbor, USA; Cat. No. 101500). Specificity controls for CB1 receptor detection were performed through preincubation of anti-CB1 rabbit antibodies with the corresponding specific blocking peptide (Cayman Chemical, Ann Arbor, USA; antibody/peptide, 1/5).

#### Western Blot Analysis of extracellular-regulated kinase (ERK)1/2

25 µg protein was separated using electrophoresis and blotted onto PVDF membrane as described for CB1 and FAAH. The blots were incubated with anti-phospho-p44/42 MAPK (pERK1/2) (1∶1000, New England Biolabs GmbH, Frankfurt, Germany) at 4°C over night in 5% non-fat dry milk solution, followed by HRP-linked anti-rabbit and developed by chemiluminescence. Membranes were then incubated with anti-p44/42 MAPK (ERK1/2) (1∶2000, New England Biolabs GmbH, Frankfurt, Germany) at 4°C over night and HRP-linked anti-rabbit and developed as described above.

#### Quantification of Western Blots

For Western Blot analysis proteins were quantified using a densitometer. The band density was determined by computer program *MCID Core* (GE Healthcare Niagara Inc., St. Catharines, Ontario, Canada). CB1 receptor and FAAH levels were plotted as quantitative densitometry analysis of signals corrected on the basis of ß-Actin content. The value of active pERK1/2 was normalized to the amount of total ERK1/2 in the same sample. Values were expressed as arbitrary units.

### Statistical Analysis

Differences between the Wistar and Fischer strains were analyzed by two-way ANOVA (pERK stimulation), two-way repeated measure ANOVA (percentage SCM reduction by SR, distance traveled after WIN injection), one-way repeated measure ANOVA (SR administration) or by Student's t-tests (behavioral tests and Western blots for FAAH and CB1). For post-hoc testing the Student-Newman-Keuls test was used. During PR testing, one Wistar and one Fischer rat did not reach the lever press criterion during training and had to be excluded from PR testing.

All data are expressed as means ± SEM. The overall level of statistical significance was defined as p<0.05, a significance level of p<0.1 was considered a statistical trend.

## Results

### Behavioral Testing

#### Limited SCM intake

**Figure 1:**
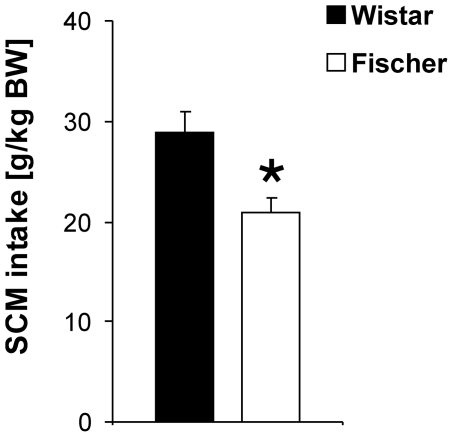
SCM intake in Wistar and Fischer rats in a limited access paradigm. Fischer rats consumed significantly less SCM than Wistar rats. Values are expressed as means ± SEM (p<0.05 is indicated by asterisks).

Differences between the two rat strains were detected for SCM intake (Figure 1). Wistar rats consumed significantly more SCM during the 15 min testing period than animals from the Fischer strain (p = 0.002) (Fischer: n = 12, Wistar n = 12).

#### PR testing

**Figure 2:**
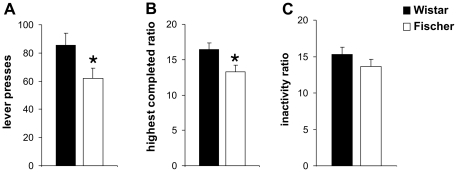
PR testing in Wistar and Fischer rats. A significant decrease in total lever presses (A) and (B) highest completed ratio was found in rats from the Fischer strain compared to Wistar rats. No significant strain differences were detected regarding the inactivity ratio (C). Values are expressed as means ± SEM (p<0.05 is indicated by asterisks).

Significant differences between animals from the Fischer and the Wistar strain were observed in PR testing (Figure 2). The number of total lever presses (p = 0.04) and the highest completed ratio (p = 0.04) were significantly lower in Fischer rats compared to the Wistar strain. However, no differences between the two rat strains were detected for the inactivity ratio (‘break point’) (p>0.05) (Fischer: n = 11, Wistar n = 11).

#### PAS

**Figure 3:**
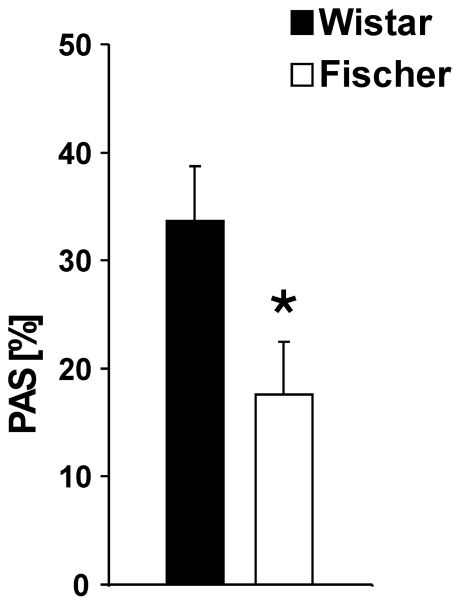
PAS in Wistar and Fischer rats. A significant lower percentage PAS response was detected in Fischer rats compared to Wistar rats, indicating a lower inhibitory effect of the conditioned odor on the startle magnitude in Fischer rats. Values are expressed as means ± SEM (p<0.05 is indicated by asterisks).

The PAS response was found to be significantly lower in rats from the Fischer strain compared with Wistar rats (p = 0.03), indicating the conditioned odor-cue induced a lower percentage reduction in ASR magnitude in Fischer than Wistar rats (Fischer: n = 12, Wistar n = 12) (Figure 3).

### Pharmacology

#### WIN effects on locomotor activity

**Figure 4:**
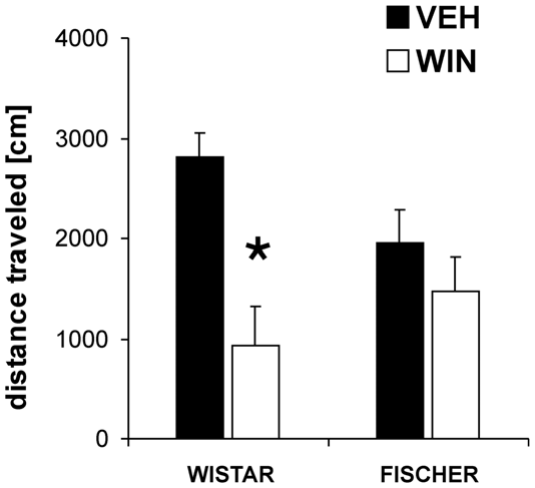
Strain specific effects of the cannabinoid receptor agonist WIN 55,212-2 (WIN) on locomotor activity. Wistar and Fischer rats did not differ significantly in their basic locomotor activity. However, an acute injection of WIN (2 mg/kg) had a stronger inhibitory effect on distance traveled in Wistar than in Fischer rats. Values are expressed as means ± SEM (p<0.05 is indicated by asterisks).

Statistical analysis for the effects of WIN on locomotory activity revealed a sigificant interaction effect for the factors treatment and strain (F_1,14_ = 10.9, p<0.05). Post-hoc analysis indicated no significant differences between Wistar and Fischer rats in general locomotor activity (p>0.05). However, the two strains differed significantly in their behavioral response to WIN. The acute injection of WIN significantly reduced locomotor activity in Wistar (p<0.001), but not in Fischer rats (p>0.05) (Figure 4) (Fischer: n = 8, Wistar n = 8).

#### Effects of SR on SCM intake

**Figure 5:**
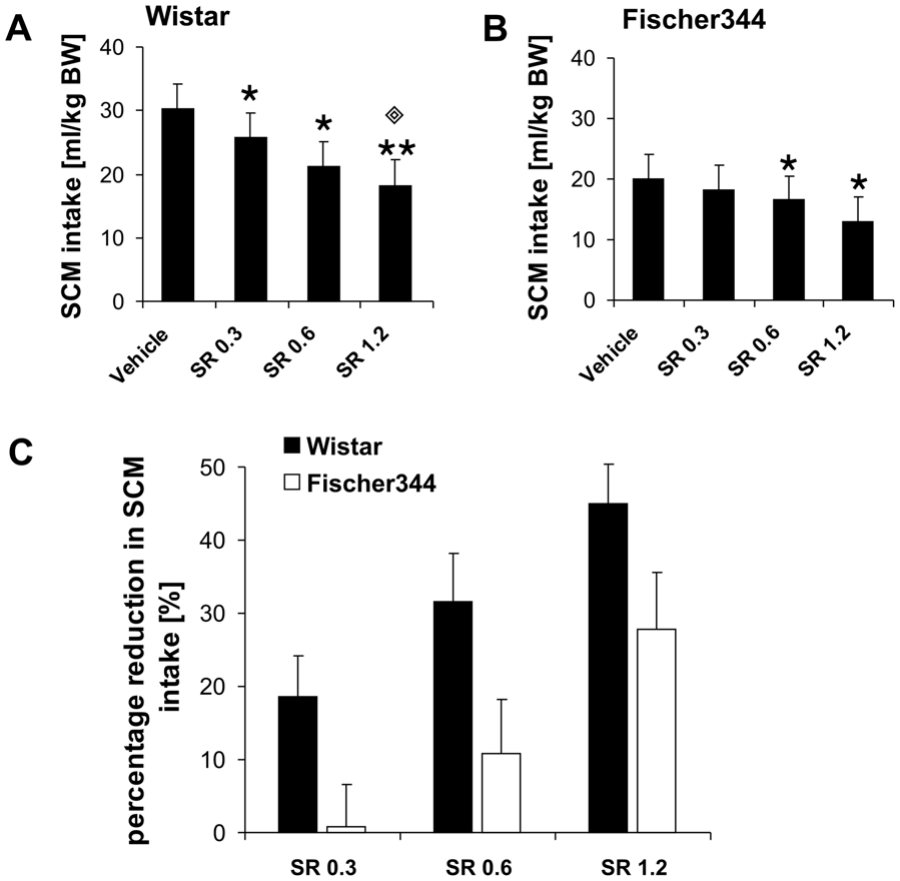
Strain specific effects of SR141716 (SR) on SCM intake. SCM intake in Wistar (A) and Fischer rats (B), as well as percentage reduction of SCM consumption (B) in both strains (C). All three concentrations of SR significantly reduced SCM intake in Wistar rats (A) (p<0.05 is indicated by one asterisk; p<0.001 is indicated by two asterisks). In addition, SCM intake in Wistar rats after treatment with 1.2 mg/kg SR was also significantly reduced compared to treatment with the lowest dose (p<0.05 is indicated by one diamond). In Fischer rats only the two highest concentrations of SR induced a significant attenuation of SCM intake compared to vehicle treatment (B) (p<0.05 is indicated by one asterisk). The percentage inhibition of SCM intake (C) induced by SR was overall significantly stronger in the Wistar strain compared to Fischer rats. Values are expressed as means ± SEM.

Treatment with the CB1 receptor antagonist/inverse agonist SR reduced SCM intake dose-dependently in both Wistar (F_3,27_ = 11.2, p<0.05) and Fischer rats (F_3,30_ = 5.3, p<0.05) (Figure 5). Post-hoc analysis revealed that all three doses of SR decreased SCM intake in Wistar rats compared to vehicle treatment (SR 0.3: p = 0.044, SR 0.6: p = 0.002, SR 1.2: p<0.001). Furthermore, the highest dose SR differed also significantly from the 0.3 mg/kg concentration (p = 0.005) (Figure 5 A).

In the Fischer strain only the two highest concentrations of SR significantly reduced SCM intake compared to vehicle injections (SR 0.6: p = 0.041, SR 1.2: p = 0.006), whereas no effect was found for the lowest SR dose (p>0.05) (Figure 5 B).

Similar results could be detected for the percentage reduction in SCM intake. Here an overall strain effect was found (F_1,38_ = 8.1, p<0.05) for the inhibition of SCM consumption by SR between Fischer rats and the Wistar strain (Figure 5 C).

### Molecular Analysis

#### Protein levels of CB1 and FAAH

**Figure 6:**
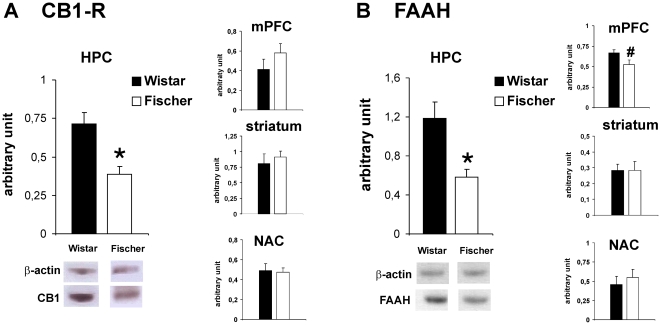
CB1 receptor (A) and FAAH (B) protein levels in different brain regions of Wistar and Fischer rats. Lower protein levels of the CB1 receptor (CB1-R) and FAAH were detected in the HPC of Fischer rats compared to the Wistar strain. Additionally, a trend for lower FAAH protein levels was observed in the mPFC of Fischer rats. No differences were observed for FAAH and CB1-R in other brain regions tested. Values are expressed as means ± SEM (p<0.05 is indicated by asterisks; p<0.1 is indicated by #).

CB1 receptor protein levels were significantly lower in the HPC of Fischer rats compared to animals of the Wistar strain (t_11_ = 3.7; p = 0.004) (Figure 6 A). No differences between the two rat strains were detected in the mPFC (t_11_ = −1.2; p>0.05), striatum (t_11_ = −0.6; p>0.05) and NAC (t_11_ = 0.2; p>0.05) (data not shown; Fischer: n = 6, Wistar n = 6).

The same brain regions as for CB1 receptor analysis were used for FAAH western blotting (Figure 6 B). No strain differences were detected in the striatum (t_11_ = 0.02; p>0.05) and NAC (t_11_ = −0.1; p>0.05) (data not shown). However, a significantly lower FAAH expression was found in the HPC of Fischer rats compared to the Wistar strain (t_11_ = 3.3; p = 0.008) (Figure 5 B) and a trend (t_11_ = 2.1; p = 0.06) for reduced FAAH levels was seen in the mPFC of Fischer rats (values mPFC: Wistar: 0.64±0.03; Fischer: 0.49±0.05) (Fischer: n = 6, Wistar n = 6).

#### pERK1/2 Stimulation

**Figure 7:**
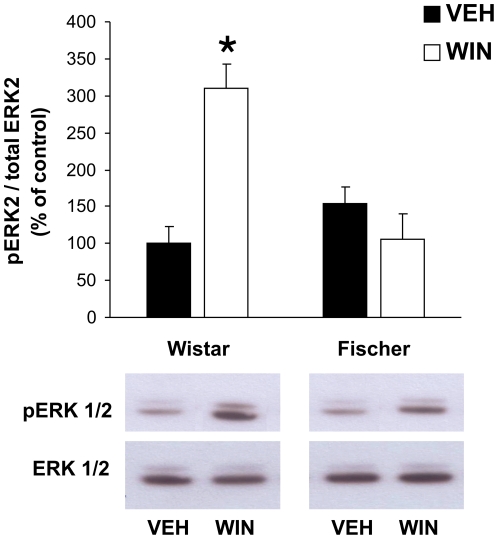
Cannabinoid-induced ERK phosphorylation in the HPC of Wistar and Fischer rats. Data for pERK2 were normalized to the optical densities of total ERK2 in the same samples and are expressed as percentage of control. Acute administration of the cannabinoid agonist WIN 55,212-2 (WIN) significantly stimulated ERK2 phosphorylation in Wistar rats (p<0.05 is indicated by asterisks), whereas no such stimulatory effect could be observed in the Fischer strain. Values are expressed as mean ± SEM.

Expression of pERK1/2 was measured by Western Blot analysis, after stimulation with the CB1 receptor agonist WIN in the HPC (Figure 7), since this was the only brain region where different CB1 receptor protein levels were discovered between the strains. Although the expression of ERK1 and ERK2 was found to be similar, the signal was always much stronger for pERK2 than for pERK1, which was below the detection threshold in some experiments. Similar low expression of pERK1 has been described by other studies after cannabinoid treatment [40]. Therefore, only values of pERK2 were used for statistical analysis. Statistical analysis revealed a significant interaction effect (F_1,20_ = 7.01; p<0.05) for the factors strain and treatment. Post-hoc analysis indicated that acute WIN administration significantly increased pERK2 expression in Wistar rats compared to vehicle treated controls (p = 0.007) but this WIN-induced increase was absent in Fischer rats (p>0.05) (Fischer: VEH: n = 6, WIN: n = 6; Wistar: VEH: n = 6, WIN: n = 6).

## Discussion

The aim of the present study was to examine possible differences in reward sensitivity towards a palatable food reward in Fischer and Wistar rats. In addition we examined if changes in ECB signaling might be involved in strain specific differences in reward-related behavior. We detected lower reward sensitivity in Fischer rats towards a food reward compared to the Wistar strain, for consummatory, motivational as well as hedonic reward aspects alike. These distinctive behavioral alterations were paralleled by basic changes in the ECB system, with Fischer rats expressing lower protein levels of the CB1 receptor and FAAH, as well as a lower ligand-induced stimulation of the CB1 receptor. Accordingly, Wistar rats showed a stronger behavioral response towards acute applications of the CB1 receptor antagonist/inverse agonist SR and the cannabinoid agonist WIN.

### Reward sensitivity

Fischer rats were found to express considerably lower reward sensitivity towards a palatable food reward than Wistar rats. For the assessment of reward sensitivity, consummatory, hedonic as well as motivational aspects of a food reward (SCM) were examined in Fischer and Wistar rats. Wistar rats consumed significantly more SCM during a limited access paradigm than Fischer rats. These findings are not related to a higher anxiogenic state or novelty-induced hypophagia in Fischer rats, since all animals were habituated repeatedly to the SCM and the complete testing procedure. Similar results were also reported in a previous study by Tordoff et al. [31], in which Wistar rats (vendor: Charles River) had a higher intake (but not preference) of polycose, saccharine and sucrose solutions (especially for lower concentrations) during a 48-h test period compared to Fischer rats (vendor: Teconic), although in this study animals were single housed and not habituated to the liquids before testing. Notably, Fischer and Wistar rats were found to express a similar number of fungiform taste papillae on the tongue, indicating no differences in their abilty to detect sweet tastants and did also not differ in their normal food intake (g/kg body weight) [31].

Besides the effects in consummatory behavior, we also observed strain differences in motivational behavior during PR testing. Wistar rats performed more lever presses and reached a higher last ratio compared to Fischer rats. Interestingly, no significant differences were detected in the inactivity ratio (no response for over 2 min) which was taken as a measure for the ‘break point’ in responding. These findings are partially consistent with an earlier study, showing that Fischer and Wistar rats did not differ significantly in “break points” for food reward [13]. However, no other parameters beside the break point (inactivity interval of 10 min) have been recorded in this specific study (e.g. total number of lever presses), since PR sessions ended as soon as the break point criterion was reached. We recently demonstrated that these different variables (break point and total lever pressing or highest completed ratio) measure different aspects during PR testing, when animals are allowed to continue lever pressing after the first “break” in responding [29], [30]. Aside from motivational aspects, the aversiveness of the progressive increase requires lever pressing and the frustration induced by the unexpected omission of reward also contribute to this first break in perseveration, but do not affect later responding. Another difference between the Freeman study and our present experiments is the diet, for which our animals were held *ad libitum* and not food restricted. A stronger motivation to obtain food after restricted feeding might attenuate differences in reward-related behaviors between the two strains.

We also examined possible rat strain differences in hedonic reward processing with the PAS paradigm. The startle probe procedure serves as a very effective means for assessing emotions, since the ASR can be modulated in humans and rats by the organism's ongoing motivational state. The decrease of the ASR amplitude, if elicited in a pleasant emotional context (e.g. the appetitive conditioned odor), serves as a cross-species model to measure reward related affect [28], [32]. The PAS paradigm offers a completely new approach for investigating the neural mechanisms of reward, since it measures the reduction of an aversive reflex, instead of reinforcing or increasing certain behaviors [28]. Fischer rats had a significantly lower percentage PAS, compared to Wistar rats. Although the appetitive conditioned odor-cue reduced the aversive startle reflex in both rat strains, this inhibitory effect was found to be much more pronounced in Wistar than Fischer animals, and therefore indicating a stronger hedonic effect of the odor-cue in the Wistar strain.

Alltogether, our findings indicate that Wistar rats express a higher reward sensitivity towards a palatable food reward compared to Fischer rats, although the response towards primary frustrating events appears not to differ between the strains.

### Molecular and pharmacological differences regarding ECB functionality

Consistent with previous studies [33]–[35], CB1 receptor antagonism significantly decreased the intake of a palatable food reward in both rat strains, however, Fischer rats were found to be less sensitive towards the inhibitory effects of SR on consummatory behavior than the Wistar strain. In Wistar rats, the lowest dose of SR (0.3 mg/kg) already induced a significant decrease in SCM intake, whereas this dose had no effect in Fischer rats. Additionally, the highest dose of SR induced stronger behavioral effects in Wistar than in Fischer rats. The percentage inhibitory effects of SR were therefore more distinct in Wistar than Fischer animals.

Similar strain effects were detected for the inhibitory effects of a high dose of WIN on locomotor activity, which were found to be much more pronounced in Wistar rats compared to Fischer rats.

These findings are in line with our observations on significantly lower protein expression levels of the CB1 receptor and the AEA degrading enzyme FAAH, in particular in the HPC of Fischer rats compared to the Wistar strain. Additionally, we observed a trend for decreased FAAH levels in the mPFC of Fischer rats. No significant differences were found in other brain regions examined in the present study, such as striatum and NAC. This is the first study demonstrating strain differences regarding CB1 receptor expression levels. Although levels of ECB ligands such as AEA were not examined in the present study, the fact that two important components of the brain ECB system are expressed to a lower extent in the HPC of Fischer rats, might indicate a basic lower ECB tone in these animals. Both CB1 receptors and FAAH are very abundant in the HPC [36], [37], which might explain why the strongest strain differences for CB1 receptor expression were discovered specifically in this region. Although no differences were observed for other reward-related brain regions (NAC, mPFC and striatum) in CB1 receptor protein levels, it is still possible that the functionality of the receptor or the endocannabinoid levels differ between the rat strains, which needs to be adressed in future studies. The strong decrease in hippocampal expression of important components of the ECB system in Fischer rats was paralleled by a lower cannabinoid-stimulated ERK activation in this region. WIN-induced stimulation of the CB1 receptor was found to activate ERK phosphorylation in the HPC to a much stronger extent in Wistar rats compared to the Fischer strain. That cannabinoid agonists are able to activate ERK in the HCP has been demonstrated before *in vitro* as well as *in vivo* [38]–[40]. This activation is mediated specifically by CB1 receptors since it can be prevented by pre-treatment with SR and additionally, ERK stimulating effects of cannabinoids are absent in CB1 receptor knockout mice [40].

Finally, our findings on stronger acute behavioral inhibition of SCM intake by SR in Wistar rats compared to Fischer rats, as well as the stronger behavioral response towards the effects of the cannabinoid agonist WIN, are consistent with a lower availability of CB1 receptors and reduced stimulation of concomitant signal transduction pathways, as observed in the present study. Although SR can be classified as a CB1 antagonist, it has been well documented in various studies *in vivo* and *in vitro* that SR behaves as an inverse agonist rather than a neutral antagonist. Thus, its biochemical or behavioral effects generally are opposite to the effects of cannabinoid agonists and include among others inhibition of MAPK activity, adenylyl cyclase activity, and GTPγS binding in selected brain regions [41], [42]. It has been suggested that SR might bind preferentially to the so-called inactive R state of the CB1 receptor, thereby decreasing the activation of the signaling pathway [41], [43]. Therefore, the lower availability of CB1 receptors in the Fischer strain might well explain the reduced behavioral effects of SR in these animals, considering its inverse cannabimimetic action at the receptor. This is further supported by our finding on a much stronger behavioral response towards application of a cannabinoid agonist in Wistar than Fischer rats.

### Impact of strain-specific alterations in the ECB system on behavioral findings

Since the ECB system has been shown to be linked to various behavioral actions, especially regarding reward-related behaviors [16], [44], basic knowledge on genetic differences in the ECB system might provide a better insight into behavioral differences between individuals. The molecular findings of the present study reveal lower CB1 receptor and FAAH expression levels as well as decreased ECB signal transduction in the HPC, and to a lesser extent in the mPFC, of Fischer rats in comparison to the Wistar strain. Both regions, the HPC as well as the PFC, have been implicated in reward processing [45], [46]. Growing evidence indicates an important modulatory role of the ECB system in reward-related behavior [16], [17], which has been shown to include ECB signaling in the HPC and the PFC [47], [48]. Although the HPC is generally not considered the primary region for brain reward processing, studies have indicated the importance of this structure for food-related and appetitive behaviors [46], [49], [50]. It has been shown that access to a highly palatable diet for some weeks in rats leads to a considerable down-regulation of CB1 receptor expression in extrahypothalamic regions, such as the HPC and the NAC, implying the HPC as an important brain region for endocannabinoid signaling in the context of appetitive food intake [46]. The modulatory influence of the HPC on reward processing has been suggested to derive mainly through its close anatomical connectivity with the nucleus accumbens [49], [50].

Molecular differences observed between both rat strains in the ECB system are further supported by our findings on decreased sensitivity of Fischer rats towards cannabinoid pharmacology. It is therefore quite conceivable that the behavioral phenotype of low reward sensitivity observed in Fischer rats in comparison to Wistar rats, is related to the low expression of important ECB components and concomitant lower activation of ECB signaling pathways in the Fischer strain.

It has been reported that genomic variations in the human CB1 receptor gene (Cnr1) appear to be involved in the vulnerability towards substance abuse and addictive behaviors [51]–[54]. Furthermore, variations in the Cnr1 and FAAH genes have been reported to be associated with a differential neural response to marijuana cues in reward pathways [55].

Additional support for the involvement of genetic variations in the ECB system in behavioral differences is given by various studies using CB1 receptor knockout mice. CB1 receptor knockout mice were found to eat less than their wild type control littermates after food restriction [56], have lower break points under PR schedules of sucrose delivery [57] and show decreased sucrose preference and consummption in a free-choice procedure [57], [58]. Additionally, lower intake rates and reinforcement strength of different drugs of abuse have been reported in CB1 receptor deficient mice compared to controls, indicating a functional role of the ECB system in mediating the addictive properties of drugs of abuse [19], [59]–[61].

Notably, in our study we did not only observe a decrease in intake and motivational properties of SCM in the Fischer strain, but the hedonic value of this palatable liquid was also attenuated in Fischer rats. It is well known that reward motivation and the hedonic impact of a reward are mediated by different neurotransmitter systems. The dopaminergic system appears to be crucial for cost-benefit calculations and motivated behavior [62], [63], whereas the endogenous opioid system is suggested to mediate euphoric or hedonic aspects of reward processing [17], [64], [65]. In line with these studies it has been demonstrated that the opioid antagonist naloxone dose-dependently decreases the PAS reponse [29], but neither dopaminergic D1 nor D2 receptor antagonists were found to affect PAS [32], thereby confirming the importance of the endogenous opioid system for the evaluation of hedonic properties of rewarding stimuli. The ECB system appears to be an important modulator of motivational and hedonic aspects of reward processing alike [17], [24], [66]–[68], mainly by close interactions with the dopaminergic [69], [70] and endogenous opioid system [17], [71]. Hence the lower reward sensitivity for consummatory, motivational and hedonic properties of the food reward in rats from the Fischer strain might be related primarily to our findings on decreased ECB activity in this rat strain, although concomittant changes in the dopaminergic or endogenous opioid system can not be excluded.

Alltogether, data from various studies in humans and rodents are demonstrating the important modulatory influence of (genetic) differences in the ECB system on reward-related behavior, indicating that reduced ECB activity is linked to attenuated reward processing.

### Conclusion

The present data demonstrate pronounced differences between the Fischer and the Wistar strain in different aspects of reward-related behavior. These behavioral findings are paralleled by congential differences in the ECB system between both rat strains. Aside from the lower reward sensitivity towards a palatable food reward, Fischer rats were found to be less sensitive towards cannabinoid treatment and to express lower levels of the CB1 receptor and FAAH, mainly in the HPC. These basic differences in the ECB system between both rat strains might contribute to the differences observed in reward sensitivity. Our data indicate that Fischer rats, in comparison to Wistar rats, represent a rat strain with a strong decrease in basic reward sensitivity and might therefore serve as a suitable model to further investigate rat strain differences in reward processing. Finally, the Fischer strain might also be of use to study the consequences of low ECB signaling, in comparison to other rat strains.
